# Correction: Cañizares-Cooz et al. Voriconazole Pharmacokinetics Administered at 4 mg/kg IM and IV in Nursehound Sharks (*Scyliorhinus stellaris*) Under Human Care. *Vet. Sci.* 2025, *12*, 17

**DOI:** 10.3390/vetsci12030281

**Published:** 2025-03-18

**Authors:** Daniela Cañizares-Cooz, Daniel García-Párraga, Sonia Rubio-Langre, Teresa Encinas, Pablo Morón-Elorza

**Affiliations:** 1 Department of Pharmacology and Toxicology, Faculty of Veterinary Medicine, Complutense University of Madrid, Av. Puerta de Hierro s/n, 28040 Madrid, Spain; sonrubio@ucm.es (S.R.-L.); tencinas@vet.ucm.es (T.E.); p-moron@hotmail.com (P.M.-E.); 2 Fundación Oceanogràfic de la Comunitat Valenciana, C/Eduardo Primo Yúfera (Científic), 1B, 46013 Valencia, Spain; dgarcia@oceanografic.org; 3 Veterinary Services, Oceanogràfic, Ciudad de las Artes y las Ciencias, C/Eduardo Primo Yúfera (Científic), 1B, 46013 Valencia, Spain

## Reference Correction

There is an error in the reference order in the original publication [1]. In the Reference section, the following corrections should be made: reference number 30 should be corrected to reference number 34. Reference number 31 should be corrected to reference number 35. Reference number 32 should be corrected to reference number 36. Reference number 33 should be corrected to reference number 37. Reference number 34 should be corrected to reference number 31. Reference number 35 should be corrected to reference number 38. Reference number 36 should be corrected to reference number 39. Reference number 37 should be corrected to reference number 40. Reference number 38 should be corrected to reference number 41. Reference number 39 should be corrected to reference number 42. Reference number 40 should be corrected to reference number 30. Reference number 41 should be corrected to reference number 43. Reference number 42 should be corrected to reference number 33. Reference number 43 should be corrected to reference number 32. Reference number 44 should be corrected to reference number 45. Reference number 45 should be corrected to reference number 46. Reference number 46 should be corrected to reference number 47. Reference number 47 should be corrected to reference number 48. Reference number 48 should be corrected to reference number 49. Reference number 49 should be corrected to reference number 50. Reference number 50 should be corrected to reference number 51. Reference number 51 should be corrected to reference number 44.

The authors state that the scientific conclusions are unaffected. This correction was approved by the Academic Editor. The original publication has also been updated.
